# Harnessing RKIP to Combat Heart Disease and Cancer

**DOI:** 10.3390/cancers14040867

**Published:** 2022-02-09

**Authors:** Kristina Lorenz, Marsha Rich Rosner

**Affiliations:** 1 Institute of Pharmacology and Toxicology, University of Würzburg, Versbacher Str. 9, 97078 Würzburg, Germany; 2 Leibniz-Institut für Analytische Wissenschaften—ISAS e.V., Bunsen-Kirchhoff-Str. 11, 44139 Dortmund, Germany; 3 Comprehensive Heart Failure Center, University Hospital of Würzburg, Am Schwarzenberg 15, 97078 Würzburg, Germany; 4 Ben May Department for Cancer Research, University of Chicago, Chicago, IL 60637, USA

**Keywords:** RKIP, ERK1/2, PKA, βAR, heart failure, cancer

## Abstract

**Simple Summary:**

Two major causes of mortality in the world today are cancer and heart disease. Although these are distinct diseases, their pathomechanisms rely partially on common signaling pathways driven by kinases such as β-adrenoceptor/protein kinase A (PKA) and the mitogen-activated protein (MAP) kinase network. Furthermore, activation of these kinases can lead to both positive and deleterious effects on disease progression. Thus, the goal of therapeutic strategies should be to promote the normalization of these signaling pathways under pathological conditions. Raf kinase inhibitory protein (RKIP) represents a physiological mechanism for achieving this outcome. As a regulator of the cellular kinome, RKIP acts in its unphosphorylated form as a suppressor of metastatic cancer progression by decreasing MAPK signaling. Conversely, in its phosphorylated form, RKIP protects against heart failure by upregulating β-adrenoceptor/PKA signaling. Here we discuss how leveraging RKIP action by using selective targeting strategies has the potential for the cardio-safe treatment of cancer.

**Abstract:**

Cancer and heart disease are leading causes of morbidity and mortality worldwide. These diseases have common risk factors, common molecular signaling pathways that are central to their pathogenesis, and even some disease phenotypes that are interdependent. Thus, a detailed understanding of common regulators is critical for the development of new and synergistic therapeutic strategies. The Raf kinase inhibitory protein (RKIP) is a regulator of the cellular kinome that functions to maintain cellular robustness and prevent the progression of diseases including heart disease and cancer. Two of the key signaling pathways controlled by RKIP are the β-adrenergic receptor (βAR) signaling to protein kinase A (PKA), particularly in the heart, and the MAP kinase cascade Raf/MEK/ERK1/2 that regulates multiple diseases. The goal of this review is to discuss how we can leverage RKIP to suppress cancer without incurring deleterious effects on the heart. Specifically, we discuss: (1) How RKIP functions to either suppress or activate βAR (PKA) and ERK1/2 signaling; (2) How we can prevent cancer-promoting kinase signaling while at the same time avoiding cardiotoxicity.

## 1. Introduction

Cancer and heart disease are both progressive diseases that represent the most important unmet clinical needs in medicine today [1,2]. Cancer ranks as one of the leading causes of death in the world. The lifetime risk of contracting cancer is ~40%, and, overall, one in four people will die from cancer. The prevalence of heart failure is estimated to be ~2% [3]. While heart failure survival improved substantially until 1990, since that time the improvement has been modest and the five-year mortality rate upon diagnosis is about 50% [3]. Notably, heart failure is also diagnosed after chemotherapy in about 2.4% of the patients without previous indices of cardiac dysfunction. Since heart failure and cancer are diseases of an aging population, cancer patients treated therapeutically are more susceptible to cardiac side effects. Recent evidence suggests that certain phenotypes derived from these diseases can be interdependent [1,4,5]. Therefore, cardioprotective therapeutic strategies for cancer treatment or multi-hit strategies would be of benefit for most patients, particularly in light of our overall aging population.

In this review, we focus on RKIP and its downstream target, the extracellular signal-regulated kinases 1 and 2 (ERK1/2), two key regulators of cancer and heart disease that can be leveraged as therapeutic targets to treat these diseases while avoiding toxic side effects. Notably, both RKIP and ERK exist in at least two discrete forms, each of which acts upon different targets. In the case of RKIP, the protein assumes two conformational states triggered by a phosphorylation switch that toggles RKIP between repression versus activation of the Raf/MAP kinase and βAR/PKA signaling cascades. For ERK1/2, a specific phosphorylation leads to alternative subcellular localization that results in transcriptional versus cytoplasmic signaling. Here we discuss how utilizing therapeutic strategies to constrain RKIP and ERK signaling states could lead to beneficial MAPK and PKA signaling while minimizing cardiotoxicity and cancer progression.

## 2. RKIP

Cellular responses to external and internal stimuli are largely mediated by protein kinase signaling cascades. The importance of the kinome to the integrity of the cellular structure and function necessitates that it is maintained under exquisite regulatory control. One of the proteins that responds to changes in the cellular kinome and resets the relative activity levels of key kinase signaling cascades is RKIP (also termed phosphatidylethanolamine-binding protein 1; PEBP1), a member of the highly conserved PEBP family. RKIP regulates both MAP kinases (ERK, c-Jun N-terminal kinase [JNK], p38) and βAR/cAMP-dependent protein kinase A (PKA) signaling pathways [6,7,8]. These kinases play an important role in both growth control and cellular response to stress, and their loss or dysregulation contributes to many disease states including tumor growth and metastasis, asthma, Alzheimer’s disease, systemic inflammatory response syndrome, and heart disease [9,10,11,12,13,14,15,16].

**Figure 1:**
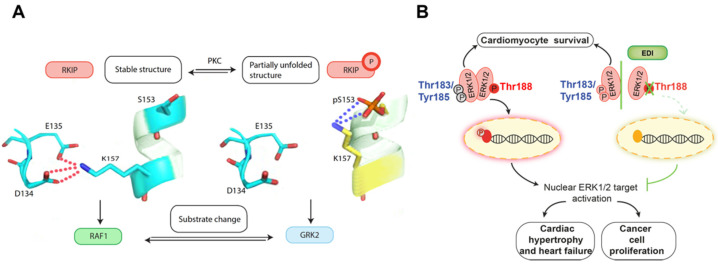
(**A**) Depicted is the phospho-theft mechanism, a novel phosphorylation-induced salt bridge theft. Specifically, the lysine located at residue 157 forms a salt bridge with two negatively charged residues on a neighboring polypeptide chain, D134 and E135. After PKC-mediated phosphorylation of RKIP at S153, the strong negative charge located just 4 residues away from K157 is enables it to effectively ‘steal’ the positively charged residue from the negatively charged residues in the neighboring salt bridge and to form a new salt bridge, resulting in a partially unfolded structure that may support the substrate change of RKIP from Raf-1 to GRK2. (**B**) pERK^T188^ autophosphorylation is induced after ERK1/2 dimerization and Gβγ binding, which triggers nuclear ERK target phosphorylation. It is causatively associated with pathological cardiac hypertrophy and cancer cell proliferation. Interference with ERK dimerization by the ERK dimerization inhibitory peptide “EDI” prevents pERK^T188^, nuclear ERK signaling, cancer cell proliferation, and maladaptive cardiac hypertrophy without interfering with ERK1/2-mediated pro-survival signals.

RKIP is a dual function protein whose role depends upon its phosphorylation state. Unphosphorylated RKIP binds to Raf-1 and inhibits its activation. Following the phosphorylation of RKIP at S153 by protein kinase C (PKC), RKIP switches its binding partner from Raf-1 to G protein-coupled receptor kinase 2 (GRK2) [11,17,18,19]. Subsequent RKIP inhibition of GRK2 activity leads to the upregulation of βAR signaling to its downstream effectors, PKA and ERK. Thus, in response to phosphorylation, RKIP acts as a molecular toggle switch for MAPK and PKA, two of the most important signaling pathways in mammalian cells (Figure 1A).

### 2.1. RKIP in Cancer

RKIP, which is often absent or downregulated in cancer, is a tumor metastasis suppressor for a variety of cancers including prostate, breast, pancreatic, lung, cervical cancers, and gliomas (reviewed in [16]). Decreased RKIP expression is negatively associated with metastatic phenotype in the majority of solid tumors and has proved to be an effective prognostic biomarker for metastasis-free survival, as well as overall metastatic risk in patients [20]. By contrast, the clinical outcome of RKIP expression in liquid tumors is complex and can differ from that in solid tumors, despite the similarities in the signaling networks regulated by RKIP (e.g., [21,22]). This is likely due in part to differences in the microenvironment that play such a key role in the metastatic progression of solid tumors.

Experimental studies conducted in a variety of tumor types, including breast and prostate, have demonstrated that exogenous RKIP overexpression in tumor cells blocks metastatic progression without significantly altering primary tumor growth. On a molecular level, RKIP functions by rewiring kinase networks to reprogram tumor cells to a nonmetastatic state [16]. In particular, RKIP is an effective inhibitor of the stress MAP kinase signaling network that promotes metastatic progression [6].

Although metastatic solid tumors commonly lack normal RKIP expression, the mechanisms by which cancer cells deactivate or eliminate RKIP prior to invasion are not well understood [16]. The general lack of mutations in RKIP suggests that transcriptional or post-transcriptional mechanisms could be largely responsible for this loss of RKIP expression. Numerous studies have implicated promoter methylation, transcriptional repression, microRNAs, and protein regulation in this process. However, attempts to induce RKIP expression in tumors lacking significant RKIP expression have been unsuccessful to date, likely due to the many mechanisms involved in the regulation of RKIP expression and stability (reviewed in [16]).

Although not as common as the loss of RKIP, significant pS153 RKIP expression has been observed in some tumors. For example, in multiple myeloma where RKIP is often overexpressed, the phosphorylated S153 form is found in about half of the patient cancers [23]. PKA, which is activated by pS153 RKIP, can act as a promoter or suppressor of tumor progression, dependent upon tumor type and conditions (reviewed in [24]). For example, PKA has been implicated in the growth and metastasis of breast and epithelial ovarian cancer but inhibits medulloblastoma. More studies need to be conducted in order to understand the effect of this phosphorylation on RKIP function in cancer and its potential efficacy as a prognostic indicator.

The net consequence is that RKIP can alter tumor cells in three discrete ways: suppressing ERK without activating PKA signaling in the wildtype state; activating ERK and PKA signaling when phosphorylated at S153 by PKC; enabling ERK activation in the absence of robust PKA signaling when depleted or deleted.

### 2.2. RKIP in the Heart

ERK and PKA signaling are also important signaling pathways in cardiac cells, and RKIP has been shown to be a major coordinator between these signaling cascades in the heart. RKIP exists mainly in its phosphorylated form in the heart and thus inhibits GRK2, which in turn leads to the activation of the most prevalent G protein-coupled receptor (GPCR) in the heart, the βAR. RKIP-mediated βAR activation subsequently leads to increased PKA signaling [25]. As the phosphorylation status of RKIP in the heart is different than in epithelial or cancer cells, RKIP does not inhibit ERK1/2 in cardiomyocytes, which is of particular relevance since ERK1/2 signaling in the heart is responsible for cardiomyocyte survival and protection from stress-induced cardiomyocyte death ([26,27]; also see Section 3.2 “ERK1/2 in the heart”). Only very high overexpression levels of wildtype RKIP, which may alter the ratio of nonphosphorylated RKIP to phosphorylated RKIP, or the overexpression of the phosphorylation-deficient RKIP mutant, RKIP^S153A^, can cause RKIP-mediated ERK inhibition in the heart. Thus, the primary function of RKIP in the heart is different from the one in epithelial or cancer cells, and the extent of ERK1/2 inhibition or PKA activation is a function of both cell-type and environmental factors.

Phosphorylated RKIP in the heart controls cardiac βAR and PKA signaling that is essential in acute situations. βAR/PKA signaling is responsible for the acceleration of cardiac contraction and relaxation that enables adaptation to hemodynamic stress situations. A pathological stress situation such as heart failure also leads to the activation of βAR and thereby stabilizes the hemodynamic situation of the patient, at least initially. However, a chronic increase in βAR and PKA signaling is detrimental [7,28,29]. Chronic βAR stimulation increases the likelihood of cardiac arrhythmias, adverse cardiac remodeling, a decline of cardiac performance, and premature death. Thus, so-called “β-blockers”, antagonists of βAR, are most commonly used to protect the heart from the noxious effects of sympathetic catecholamines in the treatment of chronic heart failure and to break the neurohumoral vicious cycle of exhausting the heart by continuous maximal stimulation [30,31]. Together, these studies show that cardiac βAR and PKA signaling can be beneficial under acute stress conditions but can lead to increased pathological disease upon chronic stimulation by catecholamines.

What is the consequence of RKIP expression in this context? Interestingly, cardiac RKIP overexpression in transgenic mice (RKIP-tg) leads to a persistent activation of βAR and PKA, as shown by the increased phosphorylation of targets downstream of βAR and PKA. Consequently, RKIP expression results in the increased speed of cardiac contraction and relaxation. However, in contrast to catecholamines, that are agonists of the βAR, the RKIP-mediated hypercontractility is well-tolerated, at least up to an age of 12–14 months, and RKIP-tg mice are protected from βAR-induced arrhythmia and cardiac remodeling such as interstitial fibrosis and apoptosis [25,28,32]. Of note, RKIP-tg mice can respond to βAR stimulation to a similar extent as wild-type mice with respect to the speed of cardiac contraction and relaxation, so that cardiac adaptation to stress situations is still feasible [25]. The reason for this difference appears to be the ability of RKIP to activate two discrete cardiac subtypes of βAR, β_1_AR and β_2_AR, in a specific manner. RKIP increases cardiac inotropy via β_1_AR and protects from arrhythmia and remodeling via β_2_AR. This cardioprotective effect of RKIP on cardiac remodeling, arrhythmia, as well as on survival was shown using mice with cardiac overexpression of RKIP (αMHC-RKIP-tg), RKIP knockout mice (RKIP-KO), and mice treated with an AAV9-RKIP gene therapy in a heart failure model of chronic left ventricular pressure overload [25]. The regulation of βAR signaling by RKIP was validated using RKIP-tg in mice lacking β_1_- or β_2_AR, respectively [25]. In sum, RKIP is an elegant example of achieving a stable and near-physiological and well-tolerated βAR/PKA activation in the cardiac context.

These data suggest that RKIP acts as a coordinator of beneficial cell signaling not only in cancer but also in the heart. However, it is not yet known how the cellular environment contributes to the cell-type specific function of RKIP and its overall beneficial signaling outcome. The RKIP switch from inhibitor to activator of PKA may be more pronounced in cardiomyocytes and may even be triggered by RKIP itself, since RKIP coordinates and is coordinated by protein kinase signaling.

### 2.3. A Novel Phospho-Theft Mechanism Underlies the RKIP Switch That Regulates PKA and ERK Signaling

Protein phosphorylation is an abundant post-translational modification that controls numerous cellular processes. In particular, phosphorylation has been implicated in the formation and dissociation of protein complexes that control protein function and mediate signal propagation within cells. A recent study of RKIP revealed a novel, evolutionarily conserved mechanism for switching protein partners, and thereby protein function, through a phosphorylation-dependent process [33]. As noted above, RKIP is a protein that has two distinct functions. In its unphosphorylated state, RKIP interacts with Raf kinase and inhibits its ability to transmit signals. Upon phosphorylation by PKC at S153, RKIP swaps its partner and binds and inhibits G protein-coupled receptor kinase 2 (GRK2) by interfering with its receptor interaction [11,17,18,19]. The release of the inhibitory clamp on GPCR, as well as MAPK signaling mediated by GRK2 and RKIP, respectively, leads to the activation of both PKA and ERK kinases. Thus, RKIP is a dual-function protein that either suppresses or activates key kinase signaling cascades dependent upon its state of S153 phosphorylation [11,18] (Figure 1A).

How does S153 phosphorylation or the substitution of two negatively charged residues at these two nearby sites lead to an exchange of RKIP partners and an alteration in RKIP function? The mechanism involves a novel phosphorylation-induced salt bridge theft that utilizes serine or threonine residues located in regions outside the binding interface and thus readily exposed to solvent. Specifically, in its wild type state, the lysine located at residue 157 forms a salt bridge with two negatively charged residues on a neighboring polypeptide chain, D134 and E135 [33]. When RKIP is phosphorylated at S153, this imparts a strong negative charge to the serine, located just four residues away from K157, enabling it to compete with negatively charged residues in the neighboring salt bridge to effectively ‘steal’ the positively charged residue and form a new salt bridge [33]. Similarly, the mutation of S153 and K157 to glutamic acid residues both breaks the initial salt bridge across two polypeptide chains in the native RKIP and disrupts local conformation in that region due to the repulsive negative charges along the α-helical polypeptide chain (Figure 1A).

There are several advantages to the mechanism by which the phospho-switch in RKIP is triggered. The classic phosphorylation association model only enables facile phosphorylation to promote protein–protein association, as residues that are involved in binding interactions would not be readily accessible to kinases. By contrast, the salt bridge theft model leaves serine and threonine residues that will undergo phosphorylation readily accessible to kinases independent of whether the protein interfaces are free or bound together. Thus, it is more likely that the phosphorylation-induced disruption of the protein interface interactions would occur by the salt bridge theft model rather than by the classic mechanism where the interface is directly phosphorylated. In addition, because there is a high frequency (two-to-three-fold enriched) of salt bridges at the interfaces of hetero-oligomeric complexes, the likelihood that a serine or threonine residue is near a charged residue will be higher at the interface, thus facilitating changes within the interface domain. The salt bridge exchange induced by nearby phosphorylation can lead to local conformational changes within a protein or can serve as a mechanism to establish or uniquely disrupt salt bridge interactions between different proteins. For RKIP specifically, this mechanism causes the disruption of the interacting polypeptide chains within the protein, enabling local conformational changes that facilitate new protein binding interactions. Ultimately this leads to the facile swapping of protein partners following PKC phosphorylation of RKIP.

Notably, the presence of S153 as a site of phosphorylation is a relatively new development in the highly conserved PEBP family, but the phospho-theft mechanism probably occurred early in the evolution [33]. A computational search for the phospho-theft motif shows a higher fraction in proteins from invertebrates (38%) relative to vertebrates (28%). The basic salt bridge structure in native RKIP, however, likely predates the salt bridge theft, as the two residues, E135 and K157, that form a cross polypeptide salt bridge are highly conserved. Interestingly, this later acquisition of a serine residue that can be phosphorylated enables more flexible interactions both within and between proteins at the vertebrate stage of evolution. As PKA signaling is a rather dominant mechanism in cardiomyocytes, responsible for the excitation–contraction cycle, this proposed theft mechanism may foster the pRKIP and the protective signaling of RKIP in the heart.

## 3. ERK1/2

Environmental signals utilize receptors, generally transmembrane proteins, to activate intracellular signaling cascades and subsequently initiate physiological responses. A central signaling cascade, in which many of those extracellular signals converge, is the MAP kinase cascade, consisting of the kinases Raf, MEK1/2, and ERK1/2. ERK1/2 are activated by various extracellular triggers such as GPCRs, integrins, and receptor tyrosine kinases, and are responsible for the induction of cellular responses such as proliferation, differentiation, and cell survival. The cascade is involved in the development and progression of many diseases including cancer, heart failure, developmental diseases, and autoimmune diseases, but are also vital for many physiological effects such as protection from cell death [34,35,36,37,38,39,40,41]. Despite the many triggers that activate this central signaling cascade, it is unclear how ERK1/2 can transmit specific and controlled cellular responses. Many hundreds of substrates of ERK1/2 are known that modulate and execute the effects of this signaling cascade. The activation of these substrates may vary depending on the type of extracellular stimuli, the availability of the scaffold proteins, and on their subcellular localization. Substrates of ERK1/2 have been identified, for example, in the cytoplasm, mitochondria, endoplasmatic reticulum, and particularly in the nucleus [35,38,42]. Thus, a tight control and understanding of the activating and modifying signals is central to direct the effects of ERK1/2 to the desired cellular outcome.

### 3.1. ERK1/2 in Cancer

MAPKs act as mediators of cellular signaling that play key roles in a multitude of biological processes, ranging from cell proliferation and differentiation to cellular stress responses and death. Among these kinase cascades, the Raf/MEK/ERK1/2 pathway is one of the most frequently dysregulated signaling pathways in cancer, particularly in solid tumors such as breast, melanoma, pancreatic, oral squamous cell, and colorectal cancers (reviewed in [43]). The cascade is involved in the regulation of cell differentiation, cell proliferation, cell survival, cell migration, and metastasis, the cause of most solid tumor death. As such, the ERK signaling pathway has been extensively reviewed (e.g., [44]). Therefore, we will focus in this review on targeting ERK in cancer and the role of ERK1/2 in the heart. Cardiotoxicity as well as drug resistance are frequent and severe limitations of prolonged treatment with Food and Drug Administration (FDA)-approved drugs that target Raf/MEK/ERK1/2 signaling in cancer [45,46,47,48,49,50,51]. ERK1/2 survival signaling is essential for the heart, especially under stress situations, and can thereby limit tumor treatments that target global ERK activity. Of note, an autophosphorylation (pERK^T188^) has been detected in cancer that does not alter overall ERK1/2 activity but has been shown to promote nuclear ERK signaling and cancer cell proliferation [26]. Interestingly, this autophosphorylation is thought to be largely associated with pathological ERK1/2 signaling, particularly in the heart [26,27,42]. The function and regulation of pERK^T188^ in cardiomyocytes is discussed in more detail below (Section 3.3). pERK^T188^ is induced in colon and lung cancer and promotes colon tumor cell growth, suggesting that this autophosphorylation may also play a role in cancer progression [26].

### 3.2. ERK1/2 in the Heart

As noted above, the role of the Raf/MEK/ERK1/2 cascade in the heart is a double-edged sword. ERK1/2 signaling is involved in adverse remodeling but is also responsible for cardiomyocyte survival and protection from stress-induced cardiomyocyte death [34,35,51,52]. Several lines of evidence demonstrate that the inhibition of Raf/MEK/ERK1/2 signaling can render the heart more vulnerable to injury [52,53,54]. Inhibitors of ERK1/2 signaling used to treat cancer have been reported to cause symptomatic heart failure, arterial hypertension, and arrhythmia [45,46,47,48,49,50,51,52]. Surprisingly, the activation of this cascade can also trigger adverse cardiac remodeling, and subsequently cardiac dysfunction and arrhythmias [36,47,55,56,57]. Thus, the suppression of Raf/MEK/ERK1/2-mediated prohypertrophic and adverse remodeling processes while preserving their survival-enhancing properties are clearly warranted, but still remain an unmet clinical need. In this context, inhibiting the hypertrophic but not the antiapoptotic signaling of ERK1/2 in the heart by, for example, targeting nuclear but not cytosolic ERK1/2 signaling could be the optimal strategy. In the following section, we will discuss the autophosphorylation of ERK1/2 at pERK^T188^ as a potential target to suppress pathological ERK1/2 signaling in the heart.

### 3.3. A Novel Modulatory Mechanism to Enable Differential ERK1/2 Signaling

Post-translational modifications and, in particular, protein phosphorylations are crucial for controlling enzymatic activity, protein conformation, and subsequent protein–protein interactions, as well as the cellular localization of certain proteins. Classically, extracellular signals are transmitted into the cell via receptor tyrosine kinases, integrins, or GPCR, and trigger the recruitment of adaptor proteins such as β-arrestin and the activation of Ras, which in turn facilitate the stepwise activation of the Raf/MEK1/2/ERK1/2 signaling cascade. The phosphorylation of the effector kinases ERK1 and ERK2 at the Thr and Tyr residues within the Thr-X-Tyr motif of their activation loop (Thr183 and Thr185 in mouse ERK2) leads to their activation and the subsequent phosphorylation of a vast array of substrates localized in all cellular compartments. These substrates include protein kinases, cell signaling, receptors, cytoskeletal proteins, and nuclear transcriptional regulators [38,57,58]. Several mechanisms are thought to contribute to the specificity of the signaling scenario despite the numerous direct and indirect targets of ERK1/2. These include the nature of the trigger, the type of receptor, the signal duration and strength, the interaction partners such as scaffold proteins, coactivated signaling pathways as direct or indirect modulators of the signaling outcome, and the cellular localization of ERK1/2 (reviewed in [38,58,59]). In this respect, while GPCR signaling induces ERK1/2 activation, the ERK1/2 signaling signature can differ depending on the type of activated G protein and the recruitment of β-arrestin [27,55,56]. For example, the activation of GPCRs coupled to inhibitory G proteins (Gi) versus stimulatory G (Gs) and Gq proteins, respectively, results in different phosphorylation patterns of ERK1/2 substrates and different signaling outcomes [55,56,57].

As mentioned above, an autophosphorylation of ERK1/2 at threonine 188 (pERK^T188^; T208 in ERK1) has been described that modulates nucleocytosolic ERK1/2 signaling. pERK^T188^ is induced by the activation of GPCR and ERK1/2 that, in turn, can result in the homo- or heterodimerization of ERK1/2. Gβγ subunits released from Gs or Gq proteins can then bind to the ERK dimer and induce the intermolecular autophosphorylation of ERK at threonine 188 [27,55,56]. Further in vitro and mouse studies revealed that ligands of GPCRs that couple to Gs or Gq (but not Gi), such as angiotensin II, endothelin I, phenylephrine, and isoproterenol, induce pERK^T188^, and that this phosphorylation site does not alter ERK activity but instead triggers the nuclear translocation of ERK. Subsequently, pERK^T188^ has been shown to trigger the activation of nuclear ERK targets such as Elk1, MSK, and c-myc, targets that not only promote cancer but are known to induce cardiomyocyte hypertrophy [60,61,62]. Indeed, pERK^T188^ is increased in human heart failure and hypertrophic mouse hearts [26,27,55,56]. Further studies showed that pERK^T188^ is only upregulated and involved in the development of pathological, but not in physiological, cardiac hypertrophy, and that it correlates with the speed of disease progression in aortic stenosis patients that suffer cardiac hypertrophy [26,27].

Altogether, pERK^T188^ appears to channel ERK1/2 signaling towards nuclear targets that can lead to pathological signaling. This autophosphorylation site is thus of great importance in controlling the ratio of cytosolic to nuclear ERK1/2 signaling and represents an important target for coordinating the signaling outcome of ERK1/2 activation in a manner that can provide protection from both heart disease and cancer (Figure 1B).

## 4. Novel Therapy Combinations That Inhibit ERK Activation and Have the Potential to Suppress Tumorigenicity without Inducing Cardiac Toxicity

As summarized above, ERK1/2 activation promotes tumor progression and metastasis. However, robust ERK1/2 inhibition by small molecule inhibitors or RKIP (unphosphorylated RKIP^S153A^), although potentially effective against cancer, also leads to the apoptosis of cardiomyocytes, cells with very limited regeneration capacity. In cancer therapy for example, increased Raf/MEK/ERK1/2 signaling is targeted by several small molecule inhibitors of the Raf, MEK or ERK, or upstream kinases or receptors, such as the epidermal growth factor receptor. While the survival of patients with certain cancer types can be extended by these inhibitors, such as in patients with advanced BRAFV600-mutated melanoma [63], cardiotoxicity and drug resistance are frequent and severe side effects of prolonged treatment with FDA-approved drugs that target Raf/MEK/ERK1/2 signaling in cancer. About 8% of BRAF and MEK inhibitor-treated cancer patients developed a significant reduction of the left ventricular ejection fraction, and about 19% developed arterial hypertension [46,64]. Thus, in the heart, ERK1/2 activity is essential for the protection of cardiomyocyte survival. However, based on the cause–effect studies of pathological cardiac hypertrophy in mice, ERK1/2 signaling is also thought to be involved in the development of human heart failure [27,55]. These findings indicate that ERK activation can be both detrimental and essential for the heart, which emphasizes a clear unmet need to develop strategies that allow either differential targeting or possibly limited activation of this signaling pathway.

There are two potential reported strategies to selectively target or partially suppress ERK signaling that may help to address the cardiotoxicity of ERK1/2 targeting in cancer therapy. First, ERK1/2 inhibition by a partial mimic of nonphosphorylatable RKIP, with respect to MAPK in tumor cells, would be one approach to treat metastatic cancer. Recently, several groups have come up with low dose multidrug strategies to target MAPK signaling in tumors (reviewed in [6]). One particular combination targeting four nodes in the MAPK network (ERK, JNK, p38) was highly effective at mimicking the anti-invasive and antimetastatic functions of RKIP both in vitro and in mice and only reduced ERK signaling by approximately 30% [6]. This broader targeting of the stress MAPK network to suppress metastasis has the potential to avoid cardiotoxicity because ERK1/2 activity is largely intact.

In addition, a strategy was developed that allows the selective targeting of pERK^T188^ by a peptide that inhibits ERK dimerization (EDI: “ERK dimerization inhibitor”). EDI does not interfere with ERK1/2 activation and antiapoptotic ERK1/2 signaling in cardiomyocytes, but instead inhibits the induction of pERK^T188^ and nuclear translocation of ERK [26]. As a consequence, ERK inhibited the activation of nuclear ERK targets and the induction of cardiomyocyte hypertrophy in vitro and in vivo. This was not only shown in cell culture using primary cardiomyocytes but also by AAV9-mediated gene therapy: AAV9-EDI reduced the development of cardiac hypertrophy, cardiac dysfunction, lung edema, interstitial fibrosis, and even cardiomyocyte apoptosis in a mouse model of chronic pressure overload. As mentioned above, pERK^T188^ also occurs in cancer. In line with this observation, EDI was also shown to effectively inhibit pERK^T188^ in cancer cells and to prevent colon cancer cell proliferation in culture [26]. Moreover, an alternative ERK1/2 targeting strategy like EDI has the potential to prevent recurrence by other proliferative cancer cell pathways, such as Akt, that might arise due to differential targeting of cytosolic and nuclear ERK1/2 targets [26].

**Figure 2:**
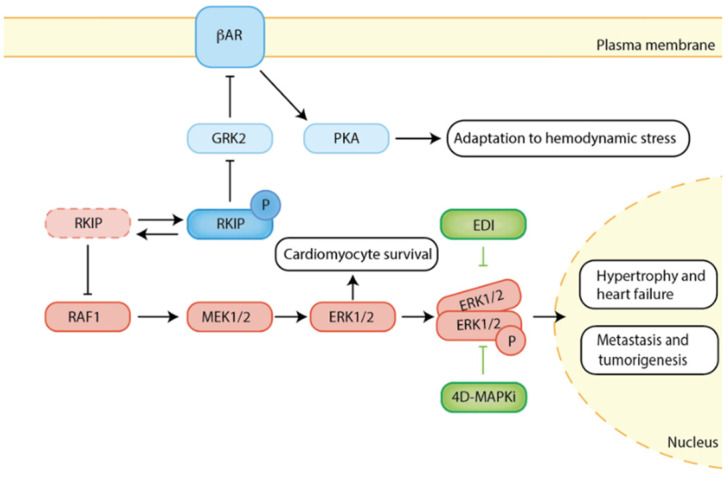
RKIP adapts to the respective cellular context and can thereby differentially affect Raf/MEK/ERK1/2 and βAR/PKA signaling depending on its phosphorylation status. RKIP results in the prevention of certain cancer types or a well-tolerated positive inotropy that can even protect from the development of heart failure in mice. The peptide EDI has potential to support the RKIP function in cancer and in heart disease by suppressing residual nuclear ERK1/2 signaling, and thus preventing cardiotoxicity, pathological cardiac hypertrophy, and heart failure, as well as supporting the inhibition of metastasis and tumorigenesis. Alternatively, the “4D MAPi”, a low dose multidrug strategy targeting MAPK signaling (ERK, JNK, p38), represents a strategy that mimics RKIP and has the potential, in particular in combination with EDI, to not only protect from cancer but also to attenuate cardiac hypertrophy and to be safe with regards to cardiotoxicity. Thus, the sophisticated targeting of the signaling cascades seems to pave the way to new multi-hit drug strategies.

Like RKIP, EDI represents a potential cardio-safe intervention strategy for heart disease. In the heart, EDI has the potential to direct βAR/ERK1/2 signaling towards improving cardiac function but does not inhibit cytosolic antiapoptotic ERK signaling that acts as an additional protective shield from cardiac adverse effects such as cardiomyocyte hypertrophy and apoptosis. In cancer, RKIP inhibits Raf/MEK/ERK1/2 signaling, thereby preventing cancer metastasis, and this can be mimicked by a low-dose multidrug combination targeting the stress MAP network. EDI could function as an additional shield to prevent increased ERK1/2 signaling from promoting cancer cell proliferation. Thus, the combination of two novel intervention strategies reveals a strong “positive” interaction and may be of interest for those patients with diseases related to heart and/or cancer (Figure 2).

## 5. Conclusions

A tight and well-balanced control of signaling cascades, such as the Raf/MEK/ERK1/2 cascade, is essential for the preservation of physiologically important cellular functions. Therefore, the normalization of the cascade’s activity under pathological conditions seems to be the perfect scenario. As noted above, RKIP is often missing in metastatic tumors, leading to excessive stress MAPK activation, but is also present in its phosphorylated form in the heart, enabling localized PKA activation. Here, we presented two different methods of PKA and ERK modulation that have the potential to suppress cancer progression and metastasis-related lethality while being cardio-safe or even protective against heart disease: (i) Treatment with partial RKIP “mimics”, such as low-dose drug combinations that target the stress MAPK network, could potentially suppress metastasis in tumors while enabling beneficial Raf/MEK/ERK1/2 and βAR/PKA signaling in cardiac cells. This could result in the prevention of cancer lethality and, at the same time, allow pRKIP in the heart to induce a well-tolerated positive inotropy and protect from the development of heart failure in mice; (ii) As EDI preserves cytosolic antiapoptotic and cardio-safe ERK1/2 signaling while suppressing nuclear ERK1/2 signaling, EDI treatment could result in protection from pathological cardiac remodeling and heart failure and the inhibition of cancer cell proliferation.

The combination of RKIP expression in the heart with kinase inhibitors such as low-dose drug combinations or EDI would allow efficient cytosolic, antiapoptotic, and cardio-safe ERK1/2 signaling while suppressing cancer progression and protecting from pathological cardiac remodeling and heart failure. These studies suggest that there are ways to generate drugs that are effective in both cancer and heart disease, or at least which do not cause cardiotoxic side effects. Thus, sophisticated targeting of the PKA and MAPK signaling cascades may pave the way to new multi-hit drug strategies for heart disease and cancer.
